# Extracellular Vesicles in *Trypanosoma cruzi* Infection: Immunomodulatory Effects and Future Perspectives as Potential Control Tools against Chagas Disease

**DOI:** 10.1155/2022/5230603

**Published:** 2022-08-17

**Authors:** Nuria Cortes-Serra, Melisa Gualdron-Lopez, Maria-Jesus Pinazo, Ana Claudia Torrecilhas, Carmen Fernandez-Becerra

**Affiliations:** ^1^ISGlobal, Barcelona Institute for Global Health, Hospital Clínic-Universitat de Barcelona, Carrer Rosselló 149-153, CEK Building. E-08036 Barcelona, Spain; ^2^IGTP Institut d'Investigació Germans Trias i Pujol, Badalona, Spain, Ctra. de Can Ruti. Camí de les Escoles, S/n, 08916 Badalona (Barcelona), Spain; ^3^CIBERINFEC, ISCIII-CIBER de Enfermedades Infecciosas, Instituto de Salud Carlos III, Spain; ^4^Laboratório de Imunologia Celular e Bioquímica de Fungos e Protozoários, Departamento de Ciências Farmacêuticas, Universidade Federal de São Paulo (UNIFESP), Rua São Nicolau, 210 Diadema, CEP, 09913-030 SP, Brazil

## Abstract

Chagas disease, caused by the protozoa parasite *Trypanosoma cruzi*, is a neglected tropical disease and a major public health problem affecting more than 6 million people worldwide. Many challenges remain in the quest to control Chagas disease: the diagnosis presents several limitations and the two available treatments cause several side effects, presenting limited efficacy during the chronic phase of the disease. In addition, there are no preventive vaccines or biomarkers of therapeutic response or disease outcome. Trypomastigote form and *T. cruzi*-infected cells release extracellular vesicles (EVs), which are involved in cell-to-cell communication and can modulate the host immune response. Importantly, EVs have been described as promising tools for the development of new therapeutic strategies, such as vaccines, and for the discovery of new biomarkers. Here, we review and discuss the role of EVs secreted during *T. cruzi* infection and their immunomodulatory properties. Finally, we briefly describe their potential for biomarker discovery and future perspectives as vaccine development tools for Chagas Disease.

## 1. Chagas Disease

Chagas disease (CD) or American trypanosomiasis is a neglected tropical disease (NTD) caused by the protozoan intracellular parasite *Trypanosoma cruzi.* The disease is widely distributed across Latin America, with an estimated 6 to 7 million individuals infected, affects vulnerable populations, and has an important impact on the health, social and economic well-being of infected individuals (WHO, 2022, accessed March 14, 2022, https://www.who.int/news-room/fact-sheets/detail/chagas-disease-american-trypanosomiasis). In the last decades, CD has become an emerging infectious disease in nonendemic regions such as Europe, North America, Japan, and Australia, with the immigration of infected people from endemic countries contributing to the spread of the infection [1–3]. Importantly, many challenges remain to control CD effectively in nonendemic areas, such as access to diagnosis (with up to 90% of cases being undiagnosed), access to treatment, and screening of pregnant women and blood banks [1, 4–6].

The transmission of the parasite occurs through contact with the infected feces/urine of blood-sucking triatomine bugs (bug vector), congenital transmission, blood transfusion, and oral ingestion of contaminated food [7]. The disease consists of two stages: the acute phase, occurring up to one week after infection and being mostly asymptomatic, and the chronic phase, with 30-40% of chronic patients manifesting cardiac and digestive symptoms [7, 8].

CD control presents multiple challenges: the parasite-host interactions are not yet completely understood; the diagnosis has several limitations; the two available treatments present several side effects and limited efficacy during the chronic phase of the disease; and there are no preventive vaccines for human or veterinary use. Additionally, there are no prognosis markers or biomarkers of therapeutic response [4, 9, 10]. In this scenario, the development of new therapeutic tools is urgently needed.

## 2. Extracellular Vesicles

Extracellular vesicles (EVs) are small particles formed by a lipid bilayer secreted by all cell types into the extracellular microenvironment and present in all body fluids [11]. Particles are divided into several subtypes, such as exosomes, microvesicles, and apoptotic bodies, according to their origin, size, and molecular composition [11–13]. Their content, which reflects the cell of origin, includes cytosolic and cell-surface proteins, nucleic acids, lipids, and metabolites. EVs mediate intercellular communication in a great variety of biological processes during homeostasis and in pathological conditions, where they act as messenger entities that deliver specific cargo to recipient cells, thereby altering their physiological status. The molecular signatures and functional properties of EVs, together with their remarkable stability in biofluids and systemic distribution, endow them with great potential to be used as biomarkers for the diagnosis and prognosis of diseases, as therapeutic vehicles for drug and gene therapy, and for developing new vaccine platforms against infectious diseases [14–18].

EVs have been described and are currently being studied in several protozoan parasites and helminths, such as *T. cruzi, Leishmania* spp., *T. brucei*, *Toxoplasma gondii*, *Plasmodium* spp., *Giardia intestinalis*, *Schistosoma mansoni*, and *Fasciola hepatica* [19–29]. In the context of the parasitic diseases that these organisms cause, EVs are known to play a major role in intercellular communication between the parasite and the host. Importantly, EVs can modulate the host immune response, increase parasite invasion, and alter the integrity and function of cells and tissues, resulting in different disease outcomes [20, 28, 30–34]. Moreover, the EV's capacity to mediate immune evasion through a broad type of mechanisms contributes to the exacerbation of infection [15]. On the other hand, EVs can also trigger protective responses by activating an immune cell effector mechanism that benefits the host, controlling parasite replication, and promoting host survival [17, 18, 23, 35, 36]. EVs released by parasites induce an immune modulatory effect and, together with their proven capacity for direct and indirect antigen presentation in the adaptive immune response [16], make them promising tools for vaccine or immunotherapy development [17, 18, 37].

Although EVs have not yet been tested as vaccines in clinical trials for parasitic diseases, several studies have demonstrated their potential. In Toxoplasmosis, EVs derived from dendritic cells incubated with *T. gondii* antigens induce a systemic immune response in mouse models. The vaccinated animals demonstrated increased survival and lower cerebral parasite burden after parasite challenge [38–40]. Another group has shown that EVs released by cells infected with *T. gondii* alter cell proliferation, causing changes in neighboring cells, which is the most likely mechanism for modulating the host's immune system [41]. In addition, using *P. yoelii* as a murine model of malaria, it has been shown that EVs generated from infected reticulocytes when administered in the presence of CpG as adjuvant elicited a potent host humoral immune response, decreased parasitemia, and protected mice against a challenge with a lethal strain of *P. yoelii* [23, 42].

EVs released in parasitic diseases contain parasite and host proteins, nucleic acids, and glycoconjugates or lipids from the parasite membrane [33, 43–46]. This characteristic, together with the fact that EVs are found in all biological fluids and present a specific molecular signature, dependent on the cell of origin, makes them interesting tools for biomarker discovery [47–49]. In *P. falciparum* and *P. vivax*, high circulating levels of EVs have been associated with the clinical symptoms and severity of the disease, showing that EV concentrations may have applications as biomarkers of malaria severity [50, 51]. In schistosomiasis, schistosomal miRNAs were detected in EVs isolated from patients before treatment. These levels decreased after treatment, indicating that EVs could be used as new diagnostic tools for patients presenting low parasitic burden, and as new biomarkers for therapeutic response [52].

## 3. Molecular Composition and Virulent Factors Associated with the Shedding of EVs by *T. cruzi* Parasite

The shedding of EVs by *T. cruzi* was first described in epimastigote form in 1979 [53]. Later, Gonçalves's group demonstrated that infective trypomastigote form from four different *T. cruzi* strains (Y, YuYu, CA1, and RA) released surface antigens bound to the particles by a spontaneous process [54]. However, it was not until 2013 that the first proteomic analysis of the *T. cruzi* secretome was performed [55]. In this study, EVs released by noninfective epimastigotes and infective metacyclic trypomastigote forms were isolated by ultracentrifugation, and two EV subtypes (larger and smaller), as well as vesicle-free fractions, were analyzed by mass spectrometry. The results showed a rich collection of proteins involved in metabolism, host-parasite interaction, signaling, nucleic acid binding, parasite survival, and virulence [55]. From then on, other proteomic studies emerged to characterize the exoproteome of trypomastigote forms [56] and to detect antigens associated with vesicles secreted by *T. cruzi* trypomastigotes [57]. Later on, another proteomic analysis of trypomastigote EVs was performed using two different strains (Y and YuYu), known to modulate the host immune responses differentially [45]. The analysis confirmed previous protein identification and showed quantitative and qualitative differences in the EV cargo of the two strains, which correlated with differences in their infection profile [45]. Recently, another study characterized the proteome and the nanomechanical properties of EVs released from trypomastigotes and epimastigotes, finding marked differences in the EV cargo between both stages [58]. The first proteomic characterization of plasma-derived EVs purified directly from a heart transplant patient with chronic Chagas disease (CCD) was performed recently, identifying both human and parasite proteins in circulating EVs [46, 59].

**Figure 1:**
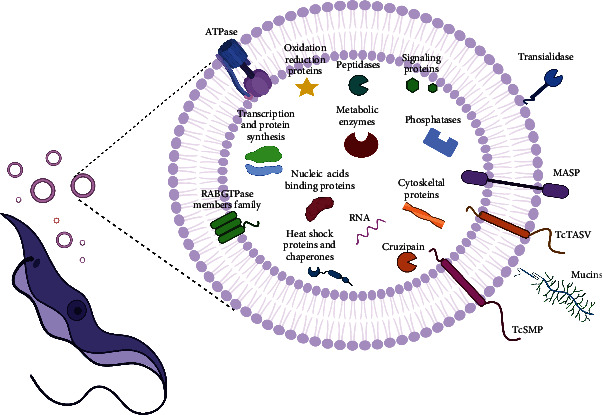
Schematic illustration summarizing the molecules identified in extracellular vesicles secreted by *T. cruzi* trypomastigotes. Several proteomic studies have identified *T. cruzi* virulence factors in EVs isolated from *T. cruzi* trypomastigotes, such as *trans*-sialidases, mucins, MASP proteins, the protease cruzipain, phosphatases, TcSMP, and TcTASV. Other molecules that can be found in trypomastigote EVs are signaling proteins, peptidases, oxidation/reduction proteins, ATPases, transcription and protein synthesis proteins, metabolic enzymes, cytoskeletal proteins, nucleic acid-binding proteins, heat shock proteins and chaperones, members of the RAB GTPase family and RNA, among others (created with http://BioRender.com).

The molecular cargo released in EVs by the different stages of *T. cruzi* parasites cultured *in vitro* is summarized in Figure 1. Among the proteins identified in EVs released to the conditioned medium by different *T. cruzi* stages, the presence of virulent factors is worth highlighting, as their expression in the parasite is fundamental for disease establishment and progression of infection. This is the case for trans-sialidases (TS), mucin, mucin-associated surface protein (MASP), cruzipain, and phosphatases, among others [19] (Table 1). These molecules, whose functions have been studied for years in the context of infection and the parasite, are involved in attachment and invasion of the host cells, protecting the parasite from complement-mediated lysis system, and may act as proinflammatory agents [60–65]. However, the function of these molecules in EVs is not yet well known and requires further investigation.

## 4. Immunomodulatory Role of EVs Derived from Trypomastigote Forms and *T. cruzi*-Infected Cells

**Figure 2:**
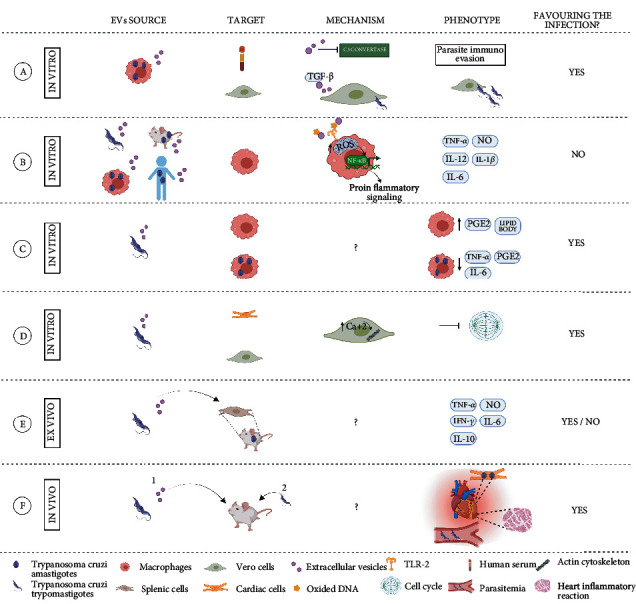
Immunomodulatory role of extracellular vesicles derived from *T. cruzi* and *T. cruzi*-infected cells. Summary of the main studies targeting the immunomodulatory effect of EVs in *T. cruzi* infection: EV source, target cell or body fluid, mechanism of action (if known), phenotype, and final effect on the infection process. (a) EVs secreted by infected macrophages can inhibit the C3 convertase, protecting the parasite against the complement system and increasing its chances of survival, and promote rapid cell invasion. (b) EVs from *T. cruzi* trypomastigotes, infected cells, infected individuals, and infected mice are recognized by uninfected macrophages via TLR2, inducing the translocation of NF-*κ*B and modulating the synthesis of proinflammatory cytokines. (c) *T. cruzi* EVs induce the formation of lipid body and PGE2 in noninfected macrophages and downregulate the synthesis of TNF-*α*, PGE2, and IL-6 in infected macrophages. (d) EVs secreted by the parasite alter cell permeability and intracellular levels of calcium in nonimmune host cells, modifying the dynamics of the cytoskeleton and arresting the cell cycle. (e) EVs secreted by trypomastigotes induce an ex vivo production of pro and anti-inflammatory cytokines in splenic cells from chronically-infected mice. (f) *In vivo* studies in mice, injected with trypomastigotes-derived EVs prior to *T. cruzi* infection, have shown an increase of circulating EVs in plasma and parasitemia, cardiac tropism, and inflammation (created with http://BioRender.com).

The early events of *T. cruzi* infection are crucial for the establishment of the disease. The parasites contain molecules that induce the host innate immune response [62]. As macrophages and other mononuclear cells are among the host's first line of defense, several research groups have focused on the study of these cells and their interaction with EVs secreted by the parasite. EVs are among the mechanisms used by the parasite to escape the immune system. It has been shown that microvesicles released by THP-1 cells, after interacting with trypomastigotes in the early stages of infection, are able to inhibit the C3 convertase, protecting the parasite against the complement system and increasing its chances of survival [66]. These authors also demonstrated that a subpopulation of microvesicles carrying transforming growth factor beta (TGF-*β*), after incubation with Vero cells, increased *T. cruzi* invasion [66] (Figure 2(a)). Previous studies have also shown that *T. cruzi* infection requires the activation of the TGF-*β* signaling pathway to increase parasite invasion in epithelial and cardiac cells and that TGF-*β* is also involved in the development of CCD cardiomyopathy, being crucial for the formation of cardiac fibrosis [67, 68].

It has been observed that EVs released by several *T. cruzi* strains (Y, Colombiana, CL-14, and YuYu) modulate the inflammatory response of macrophages via the TLR2-dependent pathway, involving the signaling pathways of mitogen-activated protein kinases (MAPK), and trigger an inflammatory response mediated by proinflammatory TNF-*α* and IL-12, IL-6, and NO [69] (Figure 2(b)). In the same line, other studies exploring the EV's contribution to the proinflammatory response of THP1 macrophages showed that vesicles isolated from the plasma of CCD patients and experimentally infected mice also triggered the synthesis of proinflammatory cytokines and oxidative and nitrosative products [70]. Interestingly, the expression levels of proinflammatory genes observed in this study depended on the patient's disease stage, being higher in CCD patients presenting symptoms than in individuals suffering the indeterminate form of the disease [70] (Figure 2(b)). Notably, an unbalanced immune response favoring a proinflammatory environment is one of the main features responsible for disease progression. In this scenario, therapies capable of preventing tissue damage or reprogramming macrophages to increase microbicide and effector functions could be a useful tool for CD treatment. More recently, Vasconcelos and collaborators showed that the viability and/or integrity of the parasite are necessary factors for the release of EVs, which trigger a proinflammatory response in the host cell *in vitro*, and may be a strategy developed by the parasite that is aimed at creating a more favorable environment for establishing infection in the host [71]. However, other studies have shown that bone marrow-derived macrophages treated with *T. cruzi*-derived EVs (strain Y) induced lipid body and prostaglandin E2 (PGE2) formation prior to infection. Twenty-four hours after *T. cruzi* infection, these EV-treated macrophages decreased the production of PGE2, TNF-*α*, and IL-6, decreasing the production of proinflammatory cytokines and oxidative and nitrosative products, which favored parasite infection and persistence (Figure 2(c)) [72].

The therapeutic potential of EVs is variable in different models and needs to be addressed carefully. Infected macrophages can also modulate the activation of other human THP-1 cells, promoting an inflammatory response [69]. Using a NF-*κ*B activation reporter CHO cell line, the authors showed that EVs secreted from infected cells induced the translocation of NF-*κ*B after interacting with TLR2 in this model. Moreover, both EVs from trypomastigotes forms and from infected macrophages altered the gene expression of proinflammatory cytokines, such as TNF-*α*, IL-6, and IL-1*β*, and signal transducer molecules, such us STAT-1 and STAT-3 in THP1 macrophages (Figure 2(b)) [69]. In another report, the mechanism of NF-*κ*B-mediated proinflammatory cytokine response was studied further and identified two proteins involved in sensing DNA damage, cGAS, and PARP1 (a DNA repair enzyme), as factors responsible for the proinflammatory phenotype induced by the parasite and infected cell-derived EVs. Oxidized DNA was detected in EVs secreted by infected macrophages and in EVs from the plasma of chronically infected mice. Interestingly, the inhibition of PARP1 decreased the overall proinflammatory response and heart inflammation of chronically infected mice, suggesting that chemical inhibitors of this enzyme could become potential therapeutic targets for CD [73]. Notably, in some of the described *in vitro* models, *T. cruzi*-infected cells released higher levels of EVs compared to noninfected cells, and these differ largely in their protein cargo [69, 74].

Little is known about the mechanisms by which *T. cruzi* EVs alter nonimmune host cells. A recent *in vitro* study showed that epithelial cells (Vero) and cardiac muscle cells (HL-1), incubated with parasite EVs, altered cell permeability and intracellular levels of calcium, which modified the dynamics of the actin cytoskeleton and arrested the cell cycle (Figure 2(d)). All together, these changes could explain the increased host-cell invasion observed in this study [75].

The role of EVs in the immune response of the chronic stage of *T. cruzi* infection has been studied less. Nogueira and collaborators studied the *ex vivo* effect of EVs secreted by different *T. cruzi* strains (Y, Colombiana, CL-14, and YuYu) when used to stimulate splenocytes from chronically infected mice. Interestingly, the immunomodulatory responses caused by the EV stimulus depended on the parasite strain. As previously reported, in other cell types, splenic cells also produced NO, TNF-*α*, IL-6, and IFN-*γ* upon stimulation with parasite EVs. However, an increase in the production of anti-inflammatory cytokine IL-10 by T and B cells was also observed, which is in contrast with the proinflammatory profile found in other studies, and reinforces the importance of IL-10 in modulating the balance between inflammatory and anti-inflammatory responses, avoiding tissue damage (Figure 2(e)) [76]. Several *in vivo* studies addressing the EV effect on the pathological features of CD have also been performed, all of which used well-established mouse models. Some of these studies have shown that animals treated with parasite-derived EVs prior to *T. cruzi* infection are distinguished by increased circulating EVs in plasma, parasitemia, cardiac tropism, and inflammation (Figure 2(f)) [28, 66, 72] . Moreover, some studies found a reduction of NO and TNF-*α* levels in plasma and a decreased production of TNF-*α* and IL-6 in spleen cells from infected animals [72]. However, some discrepancies have been observed in the mortality rates linked to EV immunization, with some studies reporting increased mortality [28] while no differences were found in others [72].

There is growing evidence that the immunomodulatory properties of EVs depend on the *T. cruzi* strain and the parasite stage [74, 76, 77]. *T. cruzi*-derived EVs from different strains present different protein cargos, which correlates with differences in the sensitivity to complement-mediated lysis, parasite invasion, infectivity, virulence, and immunomodulatory responses [45, 76]. In relation to the effect of the EVs released by host cells after interacting with different parasite developmental stages, it has been found that all *T. cruzi* stages are able to induce the release of EVs by host cells, with mammalian infective forms causing the highest release [74].

## 5. EVs as a Potential Source of New Biomarkers in Chagas Disease

The use of EVs as a new platform to identify biomarkers has been described in the last few years for different pathologies, including parasitic diseases [78]. Taking into consideration that one of the biggest challenges for CD is the lack of validated biomarkers to indicate therapeutic response and disease outcomes [59], EVs could become a promising source for developing new biomarkers in infectious diseases.

As previously mentioned, the MASP multigene family is one of the major virulence factors of *T. cruzi*. It plays a fundamental role in cell invasion and has an associated humoral immune response in CD patients. Interestingly, this response is different depending on the clinical stage of the individuals, being lower in the sera of patients presenting cardiac affection compared to sera from those suffering from the gastrointestinal form of the disease [65]. Further research showed that the EVs released by the parasite containing MASP proteins are targeted by the immune system, triggering the formation of circulating immune complexes containing anti-MASP Immunoglobulin Gs (IgGs). The EVs forming immune complexes inhibit the complement system. Interestingly, the highest percentage of inhibition appeared in the digestive group, compared to the asymptomatic and cardiac patients. Taking advantage of this particularity, these immune complexes could be used as biomarkers for the differential diagnosis or prognosis of CD, in particular in patients with digestive manifestations [61]. In the same line, microvesicles also have potential as differential diagnosis or prognosis biomarkers during CD infection. The antibodies contained in the sera from CD patients detected antigens from EVs released by host cells after interacting with the infective forms of the parasite. Interestingly, these molecules were recognized differently by patients presenting the cardiac or indeterminate phase of the disease, indicating the existence of specific markers associated with a differential diagnosis depending on the organ involved [74].

EV concentration in the body fluids of healthy individuals and patients presenting several forms of the disease has also been studied as a potential biomarker for differential diagnosis, with no clear results. While some studies did not find any statistical differences in the number of vesicles in CD patients compared to healthy controls [74], others did find differences in terms of concentration, showing that treated patients presented lower concentrations of circulating EVs than healthy donors [48]. In this study, human THP-1 cells were incubated with circulating EVs, followed by ELISA to measure cytokines and determine whether the concentration of circulating EVs was associated with differential activation of the immune system. IFN-*γ* and IL-17 showed a differential profile when compared to chronic Chagas patients and healthy controls, finding that patient samples induced a higher production of IFN-*γ*, and lower production of IL-17, a profile that could contribute to parasite persistence and tissue damage due to continuous inflammatory signaling [48] .

Two of the features of CD are chronic inflammation and oxidative stress, which are specially exacerbated in individuals suffering the cardiac form of the disease. It has been shown that microparticles generated during *T. cruzi* infection carry the host's signature for oxidative, nitrosative, and inflammatory states. Thus, EVs provide information about the disease's progression and could be useful for evaluating disease severity [70].

In a different study, a group of human and parasite proteins were identified in plasma-derived EVs from a heart transplant patient with chronic CD, while being absent in EVs from the plasma of healthy individuals. Interestingly, several human proteins and one parasite protein (pyruvate phosphate dikinase) were found to be present or upregulated before treatment and were absent or downregulated after treatment. Although these results should be interpreted with caution, as they represent a single clinical case and need to be validated in a larger cohort, they represent a proof-of-principle of the potential of this approach to discover new biomarkers of therapeutic response [46].

**Figure 3:**
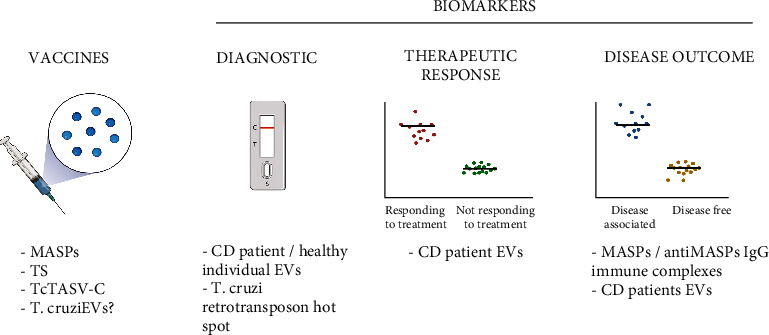
Potential role of EVs as new tools for CD prevention and control. EVs from *T. cruzi* or parasite proteins found in the EVs have been tested as vaccine antigens for Chagas disease. EVs isolated from CD patients, the retrotransposon hot spot *T. cruzi* protein, and the immunocomplexes found in CD patients are also being studied for their potential as biomarkers for diagnosis, therapeutic response, and disease outcome (created with http://BioRender.com).

Finally, EVs from *T. cruzi* are also attractive candidates for use in the serological diagnosis of CD. In an attempt to identify antigens, present in trypomastigote excreted-secreted EVs, Bautista-López and collaborators incubated trypomastigote-excreted antigens associated with EVs with affinity columns containing IgG antibodies from healthy donors, or Chagas patients with clinical symptoms. Chagasic IgG affinity resin was highly enriched in *trans*-sialidases and showed a significant enrichment in mitochondrial proteins, retrotransposon hot spot (RHS) proteins, paraflagellar rod proteins, proteases, and multiple uncharacterized proteins [57]. RHS and *T. cruzi* paraflagellar rod-3 protein were further explored for their potential as serological antigens for the diagnosis of *T. cruzi* infection, showing robust cross-reactivity with sera from patients presenting all clinical forms of CD. Interestingly, no cross-reactivity with RHS was detected when using sera from patients with other parasitic diseases, which could be relevant for the development of a new diagnostic test with high specificity [57]. The potential control strategies that could be associated with EVs secreted by *T. cruzi* or *T. cruzi*-infected cells, such as biomarker discovery and/or vaccine development, are summarized in Figure 3.

## 6. Chagas Disease Prevention: Future Perspectives of EVs as New Vaccine Antigens against *T. cruzi* Infection

Even though vaccines could be a very useful cost-effective tool for the prevention and control of *T. cruzi* infection and transmission, we are still a long way from having a beneficial vaccine for CD [79]. The lack of financial support and interest from governments and the pharmaceutical industry, together with the genetic complexity of the parasite, have contributed to the slow progress in its development [80]. Multiple attempts have been made to develop safe and effective vaccines for CD. Currently, there are two main target product profiles for developing vaccines for CD. The first one, which could be used alone or in combination with drug therapy, aims to prevent, or at least delay, the progression of cardiac and digestive manifestations in patients presenting the indeterminate form of the disease [80]. The second one is aimed at developing a preventive vaccine [81]. Unfortunately, although some of the candidates were able to induce a partial protective response, none of them showed complete protective immunity [81]. In this scenario, new approaches and ideas are needed to develop a protective vaccine for *T. cruzi* infection. Immunization with molecules delivered into EVs is an interesting possibility for exploring *T. cruzi* infection.

Interestingly, one of the protein families present in *T. cruzi*-derived EVs, which has been tested as a potential vaccine antigen, is the MASP family. Taking into consideration that MASPs play a major role in host-cell invasion, that they are one of the most important *T. cruzi* virulence factors, and that several MASP family members have predicted MHC-I and MHC-II epitopes, a synthetic MASP-derived peptide was tested as a vaccine candidate in a murine model of CD [65, 82, 83]. Mice immunized with the synthetic MASP peptide conjugated to keyhole limpet hemocyanin showed an 86% survival rate after being infected with trypomastigotes and had a much lower parasite load in the heart, liver, and spleen compared to untreated animals. Moreover, vaccinated animals produced neutralizing antibodies and developed a protective cytokine response against parasite infection. Interestingly, the vaccine engaged both humoral and cellular responses, indicating that MASP proteins are promising targets for the development of a CD vaccine [83].

Another well-known *T. cruzi* virulence factor, which is essential for the invasion process and present in EVs, is the TS family. Several investigators have tested immunization with multiple gene-encoding members of the TS family, in different vaccine platforms (bacterial and viral vectors, or as a recombinant protein) and formulations (alone, together with other *T. cruzi* glycoconjugates, and associated with adjuvants) [81, 84]. Although the results obtained showed some limitations, some vaccine formulations induced immunity in mouse models challenged with *T. cruzi*, producing antibodies, preventing the development of tissue damage, and having an impact on the mortality of infected animals [84]. In that context, TS antigens conjugated to EVs could be a different approach to developing vaccines for CD.

Another *T. cruzi* protein family secreted in EVs that has been considered for immunization is the *T. cruzi* trypomastigote alanine, valine, and serine (TcTASV-C). To evaluate the performance of TcTASV-C as a vaccine antigen, mice were vaccinated following a DNA-prime protein-boost schedule of immunization. However, when animals were challenged with a highly virulent *T*. *cruzi* strain two weeks after the final dose, the results obtained were not very promising. Although TcTASV-C-vaccinated mice showed a strong humoral response, there was a delay in the appearance of circulating trypomastigotes, and they presented lower parasitemia, exhibiting only a 30% higher survival rate than controls [60].

Finally, preliminary results have shown that mice immunization with 3 doses of EVs derived from trypomastigote forms of *T. cruzi* (Y strain), administered in the presence of Al(OH)_3_ as adjuvant, could induce some level of protection against experimental CD. Preliminary results showed that vaccinated mice presented lower parasitemia than nonvaccinated animals. However, no significant changes were observed in the survival of all animal groups. Further investigation needs to be carried out to understand which molecules are responsible for this potential protection. Moreover, experimental assays using EVs isolated from trypomastigote forms from different DTUs are needed to verify the influence of virulence factors in vaccination against experimental CD (Torrecilhas. A. C., unpublished data).

The use of different experimental models, cell types, adjuvants, doses, and vaccination regimens may also determine the development of the protective response. The key questions remaining for the development of new vaccine tools for CD are as follows: further characterization of the immune responses, development of highly efficient antigen delivery systems, animal models mimicking the chronic phase of the disease, assessment of parasite diversity and antigenic variation, study of coinfections, and use of adjuvants and new vaccination regimens together with more studies focusing on parasite tissue distribution [79].

## 7. Conclusions

In the last decade, research on the biology, function, and potential applications of EVs has grown exponentially. Even though the number of studies regarding *T. cruzi* infection and EVs is increasing every year, there is still a long way to go. Many questions remain in relation to the role of EVs in the pathogenesis of the disease and its mechanisms in pathogen-host interaction. Do the virulent factors maintain their virulent function when associated with EVs? Even though the function of these molecules has been perfectly described on the parasite surface, their specific function in vesicles is still not well known. Do the EVs secreted by the parasite or infected cells protect the host, or otherwise favor the infection? The EVs secreted by trypomastigotes favor host cell invasion and promote parasite immune evasion, increasing its survival in *in vitro* and *in vivo* studies. However, EVs secreted by the parasite, infected cells, infected individuals, and infected mice are also able to modulate macrophages, triggering a proinflammatory response against the parasite. Importantly, this inflammatory response, if unbalanced, is one of the main features responsible for disease progression in Chagas disease. Finally, it is urgent that we continue to explore the potential of EVs for antigen discovery, vaccine development, therapeutic strategies, and biomarkers, as these are among the most important challenges that we face in our efforts to control CD.

## Figures and Tables

**Table 1:** Main virulence factors associated to EVs shedding by *T. cruzi* parasites.

*T. cruzi* virulence factors	Description	EV source	References
*trans*-sialidase (TS)	*T. cruzi* is unable to synthesize sialic acid (SA) *de novo*, TS transfers *α*2-3-linked SA from host glycoproteins and glycolipids to acceptors containing terminal *β*-galactosyl residues present on the parasite surface. Avoiding lysis by serum factors and increasing invasion in the mammalian host (parasite sialylation)	T	55–58
Mucins (mucin-like glycoproteins: tGPI-mucins, eGPI or mtGPI-mucin)	Trypomastigotes (T) (i) Mucins are the main acceptors of SA in the parasite's surface (ii) Activation of the host innate immune system (iii) Induce the production of TNF-*α*, IL-12, and NO Epimastigotes (E) (i) Similar cell surface glycoprotein complex, called GP24, GP31, and GP37 (ii) Molecules maybe affect parasite migration in the vector Metacyclic trypomastigotes (MT) (i) Reported originally as the 35/50-kDa antigens (ii) Mucins from MT increased infectivity and the ability of the parasite to shed the mucins upon invasion of the host cell	T E MT	28, 55,57,76
Mucin-associated surface proteins (MASP)	MASPs proteins, considered one of the most antigenic *T. cruzi* proteins, are a very diverse protein family, with members involved in host-cell invasion and survival and multiplication of intracellular amastigotes (A)	E T A	55-58
Phosphatases	In *T. cruzi*, phosphatases present multiple roles, such as providing a source of inorganic phosphate, facilitating epimastigotes differentiation, and promoting infection	E MT T	55-58
TcSMP family	TcSMP induce calcium signaling and lysosome mobilization, facilitating the formation of the parasitophorous vacuole and parasite invasion	E MT	55
TcTASV family	Still unknown function, this family has been suggested as a potential target for intervention against *T. cruzi,* mainly due to the observation that some host-molecules trigger TcTASV-C expression *in vivo* during the infection.	T A	60
Cruzipain	The major cysteine peptidase involved in host immune evasion, cell invasion, and intracellular development	E T	28, 55, 57, 76
